# ﻿Three new species of *Sanguinoderma* (Ganodermataceae, Basidiomycota) from Southwest China revealed by morphology and phylogenetic analysis

**DOI:** 10.3897/mycokeys.118.152086

**Published:** 2025-06-09

**Authors:** Kai-Yang Niu, Xi-Jun Su, Feng-Ming Yu, Lin Li, Zong-Long Luo, Song-Ming Tang

**Affiliations:** 1 College of Agriculture and Biological Science, Dali University, Dali 671003, Yunnan, China Dali University Dali China; 2 Key Laboratory for Plant Diversity and Biogeography of East Asia, Yunnan Key Laboratory of Fungal Diversity and Green Development, Kunming Institute of Botany, Chinese Academy of Sciences, Kunming 650201, Yunnan, China Kunming Institute of Botany, Chinese Academy of Sciences Kunming China

**Keywords:** 3 novel taxa, multigene phylogeny, medical fungi, taxonomy

## Abstract

*Sanguinoderma* (Ganodermataceae) is an economically significant genus with notable medicinal value. A key diagnostic characteristic is the reddening of pores upon bruising. The genus is mainly distributed in tropical and subtropical regions, where it grows on the surface of humus or decaying tree trunks. In this study, during a survey of macrofungi in southwestern China, seven *Sanguinoderma* specimens were collected. Based on morphological characteristics and phylogenetic analysis combined with six-loci of the internal transcribed spacer (ITS), nuclear ribosomal large subunit (LSU), RNA polymerase II second largest subunit (*rpb2*), translation elongation factor 1-alpha (*tef*1-α), mitochondrial small subunit (mtSSU), and nuclear small subunit (nSSU), three new species of *Sanguinoderma* are identified and introduced: *Sanguinodermaaurantiacus*, *S.bambusae*, and *S.niger*. *Sanguinodermaaurantiacus* is characterized by a glabrous pileus with alternating concentric zones that range from black to dark orange; margins are dark red, acute, wavy, and slightly incurved when dry; the pileus context is thin, light grayish, and corky; and basidiospores are ellipsoid to subglobose ((10.0–13.5) × (8.9–10.9) μm). *Sanguinodermabambusae* grows on the forest floor in bamboo forests; it has an oval to subcircular, glabrous pileus with a lacerated margin resembling sparse petals; the pores are small (6–9 per mm) and oval to circular; and basidiospores are broadly ellipsoid ((10.1–14.4) × (8.6–11.6) µm). *Sanguinodermaniger* is characterized by a black to dark grayish orange pileus, and its context is soft and corky, turning grayish orange when dry. Cystidia are oblong to ovoid, and basidiospores are subglobose to globose ((8.9–12.6) × (8.0–10.0) µm). This study has enriched the diversity of the *Sanguinoderma* species.

## ﻿Introduction

*Sanguinoderma* (Ganodermataceae, Polyporales) was established based on multi-gene phylogenetic analysis (ITS + nLSU + *rpb*1 + *rpb*2 + *tef*1-α + TUB) and morphology, with *S.rude* Y.F. Sun, D.H. Costa, and B.K. Cui as type species (Sun et al. 2020). Previously, the species were classified within *Amauroderma* Murrill. The main characteristic distinguishing *Sanguinoderma* from *Amauroderma* is the fresh pore surface, which turns blood-red upon bruising and rapidly darkens. It is mainly distributed in tropical and subtropical regions, commonly found in saprotrophic environments, such as decaying wood or dead branches (Sun et al. 2020, 2022a, 2022b; Niu et al. 2024). A total of 26 *Sanguinoderma* species have been reported, of which 19 species have been reported in China, including the three new species reported in this study (Hyde, Wen, 2018; Hapuarachchi et al. 2019; Sun et al. 2020, 2022a, 2022b; Peres et al. 2023). *Sanguinodermarugosum* (Blume & T. Nees) Y.F. Sun, D.H. Costa & B.K. Cui, commonly known as “Xuelingzhi” in Chinese (Xu et al. 2024), is recognized as a traditional Chinese medicine in southern China. In Shennong’s Herbal Classic, the term “zhi” is divided into six categories: “red zhi, cyan zhi, yellow zhi, white zhi, black zhi, and purple zhi.” Among these, before 2020, species of *Sanguinoderma* were considered as a part of the *Amauroderma*, which was often referred to as “black zhi” in ancient Chinese texts. This category was believed to have anti-aging and longevity effects (Liu 2022).

Before the establishment of the *Sanguinoderma*, it was classified in the *Amauroderma*. In 2016 to 2017, Costa-Rezende et al. conducted a multi-gene phylogenetic (ITS, LSU, *rpb*1, and *tef1-α*) analysis of the *Amauroderma*; they found two unrelated clades, namely *Amauroderma* s.str. clades and *Amaurodermarude* clades. Unfortunately, they did not find any morphological differences between them (Costa-Rezende et al. 2016, 2017), so no further taxonomic investigation was conducted for these two clades. Until 2020, Sun et al., based on phylogenetic analysis (ITS, nLSU, *rpb*1, *rpb*2, *tef*1-α, and TUB) and morphological studies, proposed the *Amaurodermarude* clade as a new genus, *Sanguinoderma*, which can be distinguished from *Amauroderma* s.str. by its conspicuous reaction in the pore surface, which rapidly changes to blood red when bruised (Sun et al. 2020).

Since then, the taxonomic status of *Sanguinoderma* has gradually become clearer, and before its classification was fully clarified, *Sanguinoderma* had already attracted the attention of many scholars in the field of medicinal research. Notably, *Sanguinodermarugosum* has already demonstrated significant medicinal potential (Chan et al. 2015, Liew et al. 2015, Selvi et al. 2015, Seng et al. 2017, Zhang 2017, Chen et al. 2021, Lee et al. 2021; Li et al. 2021). These findings highlight the importance of further exploration and conservation of biodiversity in the southwestern region of China to unlock the full potential of these underexplored species.

Systematic investigation of *Sanguinoderma* species in southwestern China not only contributes to taxonomic research but also provides new and diverse resources for the development of novel drugs. During our field surveys in southwestern China, we collected seven specimens of *Sanguinoderma*. Based on phylogenetic analysis and morphological characteristics, these specimens were identified as three new species.

## ﻿Materials and methods

### ﻿Sample collection

Seven *Sanguinoderma* samples were collected during August 2023 and June 2024 from southwest China. The collection and documentation process for *Sanguinoderma* specimens entailed capturing photographs and recording essential information, such as habitat, altitude, collection time, and location ([Bibr B27][Bibr B38], 2025). Detailed descriptions of the morphological features were also documented. After collection, the specimens were then dried in an oven at 40–50 °C (Hu et al. 2022) and placed in self-sealing bags with an appropriate amount of silica gel to prevent moisture regain. The specimens have been deposited in the herbarium of Cryptogams, Kunming Institute of Botany, Academia Sinica (KUN-HKAS).

### ﻿Morphological observations

Macro-morphological studies were conducted following the protocols provided by Sun et al. (2022a), and the color was compared to standard colors on the colorhexa website (https://www.colorhexa.com) (accessed on March 1, 2025). Micro-morphological structures were observed from the dried specimens and photographed using a Nikon ECLIPSE Ni-U compound microscope equipped with a Nikon DS-Ri2 digital camera. Microscopic observations and color reactions were performed on slide preparations using 5% KOH, Melzer’s reagent, and Cotton Blue. Measurements were performed using Image Frame Work (version 0.9.7). At least 20 basidiospores were measured in each specimen; 5% of the measurements were excluded from each end of the range, and extreme values are given in parentheses (He et al. 2022). The following abbreviations were used: IKI = Melzer’s reagent; IKI– = neither amyloid nor dextrinoid; CB = Cotton Blue; CB+ = cyanophilous; L = mean spore length (arithmetic average of all spores); W = mean spore width (arithmetic average of all spores); Q = L/W ratio; (a) number of spores measured, (b) specimen number (Sun et al. 2020). Ultrastructures of basidiospores were observed using Scanning Electron Microscopy (SEM) at the Yunnan Academy of Agricultural Sciences, China.

### ﻿DNA extraction, PCR amplification, and sequencing

Genomic DNA was extracted from dried specimens using the Ezup Column Fungi Genomic DNA Purification Kit (Sangon Biotech Limited Company, Kunming, Yunnan, China), according to the manufacturer’s protocol. The internal transcribed spacer (ITS) regions were ampliﬁed with primer pairs ITS5 and ITS4 (White 1990); LR0R and LR5 for the large subunit of the nuclear ribosomal RNA gene (LSU) (Vilgalys and Hester 1990); and primer pairs RPB2–5F and RPB2–7CR (Liu et al. 1999) were used to amplify the second subunit of RNA polymerase II (*rpb*2). The translation elongation factor 1-α gene (*tef*1-α) was ampliﬁed using primer pairs EF1–983F and EF1–1567R (Rehner and Buckley 2005). The small subunit mitochondrial rRNA gene (mtSSU) was amplified with the primer pairs MS1 and MS2 (White 1990), and the small subunit nuclear ribosomal RNA gene (nSSU) with the primer pairs PNS1 and NS41 (White 1990).

The PCR volume contained 1 µL of each primer, 1 µL of extracted DNA, 9.5 µL of ddH_2_O, and 12.5 µL of 2 × EasyTaq PCR SuperMix (Sangon Biotechnology Co., Kunming, China). PCR cycling schedules for the four gene regions of ITS, LSU, mtSSU, and nSSU were obtained from Sun et al. (2022a). PCR cycling schedules for the two-gene regions of *tef*1-α and *rpb*2 were obtained from Niu et al. (2024). PCR amplicons were sent to Sangon Biotech (China) for sequencing. DNA sequences were assembled and edited in Sequencher (version 4.1.4), and the assembled DNA sequences were deposited in GenBank (Table 1).

**Table 1.** Taxa information and GenBank accession numbers of the sequences used in this study. The sequences generated by the new species sequences are shown in bold after the species name, the type specimens show “T” after the number, and - refers to the data unavailability.

Species	Voucher	ITS	LSU	*rpb*2	*tef*1-α	mtSSU	nSSU	References
***S.aurantiacus***	**HKAS 144479**	**PQ663248**	-	**PV168184**	**PV168190**	**PQ660762**	**PQ660750**	**This study**
	**HKAS 144478** ^T^	**PQ663249**	**PQ660756**	**PV168183**	**PV168189**	**PQ660763**	**PQ660751**	**This study**
*Sanguinodermabataanense*	Dai 10746	MK119832	MK119911	MK121511	MK121581	MZ352801	MZ355267	Sun et al. 2020, 2022a
	Cui 6285	MK119831	MK119910	MK121537	MK121580	MZ352793	MZ355238	Sun et al. 2020, 2022a
	Dai 7862	KJ531658	-	-	-	-	-	Li and Yuan 2015
***S.bambusae***	**HKAS 144474^T^**	**PQ663246**	**PQ660755**	**PV168182**	**PV168188**	**PQ660760**	**PQ660749**	**This study**
	**HKAS 144473**	**PQ663247**	-	-	-	**PQ660761**	-	**This study**
*S.concentricum*	HKAS 135640 ^T^	PP951682	PP951731	PP998313	PP998318	PP988462	PP982317	Niu et al. 2024
	HKAS 135641	PP951683	PP951732	-	PP998319	PP988463	PP982318	Niu et al. 2024
*S.dehongense*	HKAS 135636 ^T^	PP947806	PP951727	PP998309	PP998314	PP988458	PP982313	Niu et al. 2024
	HKAS 135637	PP951679	PP951728	PP998310	PP998315	PP988459	PP982314	Niu et al. 2024
*S.elmerianum*	HMAS 133187	MK119834	MK119913	-	-	MZ352824	MZ355234	Sun et al. 2020, 2022a
	Dai 20634	MZ354875	MZ355082	-	MZ221724	MZ352821	MZ355148	Sun et al. 2022a
	Cui 8940	MK119833	MK119912	-	-	MZ352812	MZ355305	Sun et al. 2020, 2022a
*S.flavovirens*	Cui 16935^T^	-	MK119914	MK121532	MK121582	MZ352811	MZ355254	Sun et al. 2020, 2022a
*S.guangdongense*	Cui 17259^T^	MZ354877	MZ355123	MZ358834	MZ221726	MZ352816	MZ355139	Sun et al. 2022a
	Dai 16724	MZ354876	MZ355117	MZ358833	MZ221725	MZ352815	MZ355271	Sun et al. 2022a
	Dai 20419	MZ354890	MZ355083	MZ358835	MZ221727	MZ352818	MZ355155	Sun et al. 2022a
*S.infundibulare*	Dai 18149^T^	MK119847	MK119926	MK121529	MK121597	MZ352790	MZ355239	Sun et al. 2020, 2022a
	URM 450213	MK119849	MK119927	-	-	MZ352792	MZ355252	Sun et al. 2020, 2022a
	Cui 17238	OM780277	-	MZ358837	MZ221729	MZ352800	MZ355149	Sun et al. 2022a
*S.laceratum*	A5	MG383652	-	-	-	-	-	Sun et al. 2022b
	Cui 8155^T^	NR174040	MK119928	-	-	MZ352810	-	Sun et al. 2020, 2022a
*S.leucomarginatum*	Dai 12264	OP700311	OP700344	OP696845	OP696857	OP703259	OP700325	Sun et al. 2022b
	Dai 12377^T^	OP700312	OP700345	OP696846	OP696860	OP703260	OP700326	Sun et al. 2022b
	Dai 12362	KU219986	KU220009	OP696847	OP696858	OP703261	OP700327	Song et al. 2016
*S.longistipitum*	Dai 20696^T^	MZ354881	MZ355084	-	MZ221732	MZ352822	MZ355145	Sun et al. 2020, 2022a
	Cui 13903	MZ354882	MZ355114	MZ358839	MZ221733	MZ352809	MZ355301	Sun et al. 2020, 2022a
	Dai 16635	MZ354883	MZ355120	MZ358840	MZ221734	MZ352802	MZ355260	Sun et al. 2020, 2022a
*S.melanocarpum*	Dai 18512	MZ354888	MZ355118	-	MZ221735	MZ352794	MZ355313	Sun et al. 2020, 2022a
	Dai 18603^T^	MZ354889	MZ355113	MZ358841	MZ221736	MZ352796	MZ355281	Sun et al. 2020, 2022a
*S.microporum*	Cui 13851^T^	MK119854	MK119933	MK121512	MK121602	MZ352797	MZ355270	Sun et al. 2020, 2022a
	Cui 14022	MK119856	MK119935	MK121515	MK121604	MZ352798	MZ355298	Sun et al. 2020, 2022a
	Cui 16335	MK119857	MK119936	MK121514	MK121605	OP703262	OP700328	Sun et al. 2020, 2022a
	Cui 14001	MK119855	MK119934	MK121513	MK121603	OP703263	OP700329	Sun et al. 2020, 2022b
*S.microsporum*	Dai 16726^T^	-	MZ355119	-	MZ221737	MZ352795	MZ355272	Sun et al. 2022a
	Cui 13897	MZ354878	MZ355127	-	MZ221739	MZ352804	MZ355300	Sun et al. 2022a
	Cui 13901	MZ354879	MZ355121	-	MZ221738	MZ352803	MZ355299	Sun et al. 2022a
***S.niger***	**HKAS 144475**	**PQ663243**	**PQ660752**	-	**PV168185**	**PQ660757**	-	**This study**
	**HKAS 144476**	**PQ663244**	**PQ660753**	-	**PV168186**	**PQ660758**	**PQ660747**	**This study**
	**HKAS 144477^T^**	**PQ663245**	**PQ660754**	-	**PV168187**	**PQ660759**	**PQ660748**	**This study**
*S.ovisporum*	HKAS 135638^T^	PP951680	PP951729	PP998311	PP998316	PP988460	PP982315	Niu et al. 2024
	HKAS 135639	PP951681	PP951730	PP998312	PP998317	PP988461	PP982316	Niu et al. 2024
*S.perplexum*	Cui 6496	KJ531650	KU220001	MK121538	MK121583	MZ352825	MZ355263	Li and Yuan 2015; Sun et al. 2022a
	Cui 6554	MK119835	MK119915	MK121540	MK121585	MZ352826	MZ355264	Sun et al. 2020, 2022a
	Dai 10811	KJ531651	KU220002	MK121539	MK121584	MZ352827	MZ355302	Li and Yuan 2015; Sun et al. 2022a
	Wei 5562	KJ531652	-	-	-	-	-	Li and Yuan 2015
*S.preussii*	HMAS 130806	OP700313	OP700346	-	-	OP703264	OP700330	Sun et al. 2022b
	Dai 20438	OP700314	OP700347	OP696848	OP696869	OP703265	OP700331	Sun et al. 2022b
	Dai 20622	OP700315	OP700348	-	OP696862	OP703266	OP700332	Sun et al. 2022b
	Dai 20624	OP700316	OP700349	-	OP696863	OP703267	OP700333	Sun et al. 2022b
*S.reniforme*	Cui 16511^T^	NR174041	MK119929	MK121531	MK121599	-	MZ355322	Sun et al. 2020, 2022a
*S.rude*	MEL 2317411	MK119842	-	MK121524	MK121592	MZ352819	MZ355306	Sun et al. 2020, 2022a
	DHCR457	MN077517	MN077551	-	MN061693	-	-	Costa-Rezende et al. 2020
	Cui 16592	MK119836	MK119916	MK121521	MK121586	MZ352924	MZ355307	Sun et al. 2020, 2022a
*S.rugosum*	Cui 16160	MK119845	MK119924	MK121520	MK121595	OP703268	OP700334	Sun et al. 2020, 2022b
	Cui 16337	MK119844	MK119923	MK121519	MK121594	OP703269	OP700335	Sun et al. 2020, 2022b
	Cui 17260	OP700317	OP700350	OP696849	OP696859	OP703270	OP700336	Sun et al. 2022b
	Cui 14033	OP700318	OP700351	OP696850	OP696864	OP703271	OP700337	Sun et al. 2022b
	Cui 8972	OP700319	OP700352	OP696852	OP696861	OP703272	OP700338	Sun et al. 2022b
	Dai 16437	OP700320	OP700353	OP696853	OP696866	OP703273	OP700339	Sun et al. 2022b
	Cui 6185	-	OP700354	OP696851	OP696867	OP703274	OP700340	Sun et al. 2022b
*S.sinuosun*	MEL 2366586	MK119852	MK119930	MK121527	MK121600	MZ352920	MZ355261	Sun et al. 2020, 2022a
	MEL 2341763^T^	MK119853	MK119931	MK121525	MK121601	MZ352820	MZ355291	Sun et al. 2020, 2022a
*S.tricolor*	Cui 18242	MZ354992	MZ355099	MZ358843	MZ221743	MZ352829	MZ355303	Sun et al. 2022a
	Cui 18292	-	MZ355101	-	MZ221742	MZ352828	MZ355273	Sun et al. 2022a
	Dai 18574	MZ354993	MZ355102	MZ358844	MZ221744	MZ352830	MZ355265	Sun et al. 2022a
*Sanguinoderma* sp. 1	Cui 11017	OP700321	OP700355	OP696854	OP696865	OP703275	OP700341	Sun et al. 2022b
	HMAS 59720	OP700322	OP700356	-	OP696870	OP703276	OP700342	Sun et al. 2022b
*Sanguinoderma* sp. 2	Cui 8795	MK119843	MK119922	MK121516	-	MZ352799	MZ355266	Sun et al. 2022b
	Dai 20582	MZ354887	MZ355085	MZ358842	MZ221741	MZ352823	MZ355156	Sun et al. 2022b
	Cui 9011	KJ531664	KU220010	MK121517	KU572504	MZ352805	MZ355237	Sun et al. 2022b
	Cui 9012	KJ531665	KU220011	MK121518	KU572503	MZ352807	MZ355269	Sun et al. 2022b
	Cui 9066	MZ354884	MZ355122	-	MZ221740	MZ352806	MZ355268	Sun et al. 2022b
*Sanguinoderma* sp. 3	Dai 16810	OP700323	OP700357	OP696855	OP696868	OP703277	OP700343	Sun et al. 2022b
	Cui 18251	OP700324	OP700358	OP696856	OP696871	OP703278	-	Sun et al. 2022b
*M.subresinosum*	Dai 18626	MK119823	MK119902	MK121507	MK121571	MZ352831	MZ355211	Sun et al. 2020, 2022a
	Cui 18262	MZ354871	MZ355088	-	-	MZ352832	MZ355258	Sun et al. 2022a

### ﻿Sequence alignment and phylogenetic analysis

The ITS, LSU, *rpb*2, *tef*1-α, mtSSU, and nSSU sequences used in this study were combined into a dataset. *Magodernasubresinosum*, which is the sister clade of *Sanguinoderma*, was used as the outgroup (Sun et al. 2022a). Sequences were aligned using the online version of MAFFT (version 7) (https://mafft.cbrc.jp/alignment/server/) (Katoh and Standley 2013) and manually adjusted using BioEdit (version 7.1.3) (Hall 1999). Ambiguously aligned regions were excluded from the analyses, and gaps were treated as missing data. AliView and PAUP (version 4.0) were used to convert the FASTA alignment file to a Nexus file for Bayesian analysis (Swofford 2003).

Based on the combined dataset, the maximum likelihood (ML) analysis was conducted using RAxML-HPC2 (version 8.2.3) (Stamatakis 2014), implemented on the CIPRES portal (https://www.phylo.org/portal2/login.action) (Miller et al. 2010), with the GTR + G model for each gene and 1,000 rapid bootstrap (BS) replicates. As no supported conflict (BS ≥ 75%) was detected among the topologies, the six single-gene alignments were concatenated using a Sequence Matrix (Vaidya et al. 2011).

Bayesian analysis was performed in MrBayes (version 3.2) (Ronquist et al. 2012), and the best-fit models of sequence evolution were estimated by MrModeltest (version 2.3) (Guindon and Gascuel 2003; Nylander 2004; Darriba et al. 2012). The selected models were HKY+G for ITS, GTR+I for LSU and nSSU, GTR+I+G for mtSSU and *rpb*2, and HKY+I+G for *tef*1-α. The Markov Chain Monte Carlo (MCMC) sampling approach was used to calculate posterior probabilities (PP) (Rannala, Yang, 1996). Bayesian analysis of six simultaneous Markov chains was run for 10,000,000 generations, and trees were sampled every 1,000 generations. The first 5,000 trees, representing the burn-in phase of the analyses, were discarded, while the remaining 1,500 trees were used to calculate posterior probabilities in the majority rule consensus tree (the critical value for the topological convergence diagnostic is 0.01).

Bootstrap support values in maximum likelihood (ML) equal to or greater than 60 and Bayesian posterior probabilities (PP) equal to or greater than 0.95 are given above the nodes. All trees were viewed in FigTree (version 1.4.0) (http://tree.bio.ed.ac.uk/software/figtree/). Edited using Adobe Illustrator CS5 (Adobe Systems Inc., United States) (He et al. 2022). The sequences derived in this study were deposited in GenBank. The final sequence alignments and phylogenetic trees are available at Figshare (doi: 10.6084/m9.figshare.28532450).

## ﻿Results

### ﻿Phylogenetic analyses

In this study, 34 new sequences were generated from seven samples, including seven of ITS, seven of mtSSU, six of LSU, five of nSSU, three of *rpb*2, and six of *tef*1-α sequences. These sequences were deposited in the NCBI database (https://www.ncbi.nlm.nih.gov/). A total of 408 sequences were used for the phylogenetic analysis, including 74 of ITS, 72 of LSU, 52 of *rpb*2, 67 of *tef*1-α, 73 mtSSU, and 70 of nSSU sequences. These sequences were derived from 78 samples representing 26 species, including the outgroup *Magodernasubresinosum* (Sun et al. 2022b). The total length of the combined dataset of six gene loci (ITS: 1–543, mtSSU: 544–1,045, LSU: 1,046–1,918, nSSU: 1,919–3,002, *rpb*2: 3,003–4,039, *tef*1-α: 4,040–4,558) is 4,558 bp including gaps.

**Figure 1.**
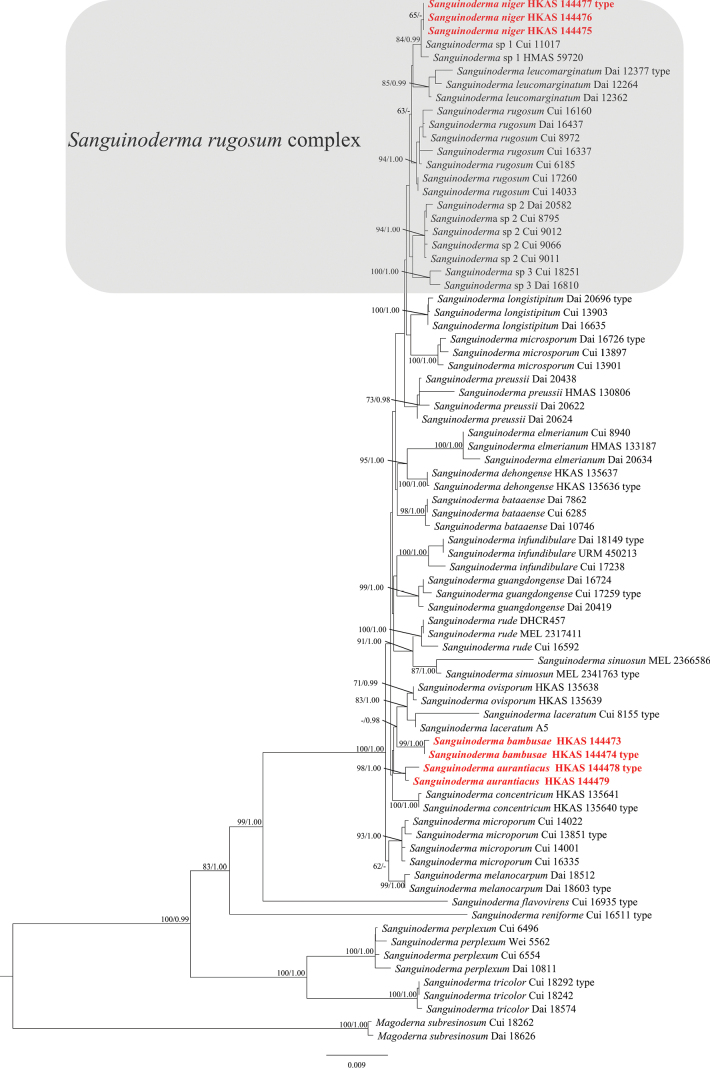
Maximum likelihood (ML) phylogenetic tree of *Sanguinoderma* based on ITS + LSU + *rpb*2 + *tef*1-α + mtSSU + nSSU. Branches are labeled with maximum likelihood (ML) bootstrap values equal to or greater than 60 and Bayesian posterior probability (pp) values equal to or greater than 0.95, shown as “ML/PP.” New species are indicated in bold red.

The RAxML analysis of the combined dataset yielded the best-scoring tree with a final ML likelihood value of -13727.303667. The matrix had 698 distinct alignment patterns, with 15.37% undetermined characteristics or gaps. Estimated base frequencies were as follows: A = 0.258667, C = 0.225588, G = 0.268657, T = 0.247088; substitution rates AC = 0.797238, AG = 3.357824, AT = 1.309244, CG = 1.351927, CT = 7.243414, GT = 1.000000, α = 0.020000; tree length: 0.340489. The best model for Bayesian analysis using MrModeltest 2.3 was HKY+G for ITS [Lset nst = 2, rates = gamma; Prset statefreqpr = dirichlet (1, 1, 1, 1)], GTR+I+G for mtSSU and *rpb*2 [Lset nst = 6, rates = invgamma; Prset statefreqpr = dirichlet (1, 1, 1, 1)], GTR+I for LSU and nSSU [Lset nst = 6, rates = propinv; Prset statefreqpr = dirichlet (1, 1, 1, 1)], and HKY+I+G for *tef*1-α [Lset nst = 2, rates = invgamma; Prset statefreqpr = dirichlet (1, 1, 1, 1)]. ML and BI analyses generated nearly identical tree topologies, with little variation in statistical support. Multi-locus phylogenetic analysis revealed that the seven *Sanguinoderma* samples were grouped into three independent clades with good support (Fig. 1), which were described as three new species: *Sanguinodermabambusae* (99/1.00) formed a sister clade with *S.ovisporum* and *S.laceratum*; *S.niger* (65/-) formed a sister clade with *S.leucomarginatu* (84/0.99); and *S.aurantiacus* (98/1.00) formed a sister clade with *S.bambusae*.

### ﻿Taxonomy

#### ﻿ Sanguinoderma aurantiacus

Taxon classificationFungiPolyporalesGanodermataceae

K.Y. Niu, S.M. Tang & Z.L. Luo sp. nov.

E832857B-8689-5054-A4E9-0558922F8755

Fungal Names: FN 572239

Figs 2 , 5a, b

##### Diagnosis.

*Sanguinodermaaurantiacus* differs from *S.bambusae* by having a funnel shape with alternating concentric zones that are black to dark orange pileus, larger pores, a thinner pileus, and basidiospores that are ellipsoid to subglobose.

**Figure 2.**
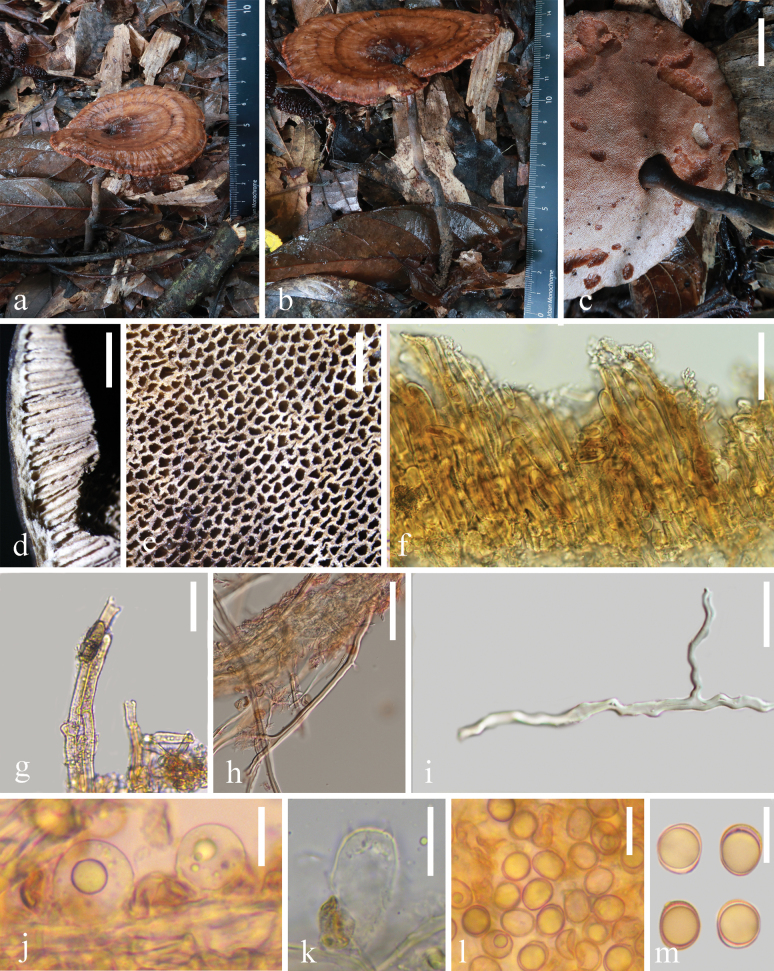
Morphological characteristics of *Sanguinodermaaurantiacus* (HKAS 144478) **a–c** basidiomata **d** pileus cross-section **e** pores **f** pileipellis **g** generative hyphae **h** skeletal hyphae **i** binding hyphae **j** basidioles **k** basidia **l, m** basidiospores. Scale bars: 1 cm (**c**); 3 mm (**d**); 1 mm (**e**); 30 μm (**f, g, h, l**); 20 μm (**i**); 15 μm (**j, k, m**).

##### Etymology.

The epithet “*aurantiacus*” refers to the dark orange pileus.

##### Holotype.

China • Yunnan Province, Dehong Prefecture, on the ground with humus, alt. 1,548 m, 24.365600°N, 98.048299°E, Kai-Yang Niu, 8 August 2023, HKAS 144478.

##### Description.

***Basidiomata*** annual, centrally to laterally stipitate, corky to woody, and hard. ***Pileus*** funnel-shaped, up to 5 cm in diameter and 5 mm thick; ***pileus surface*** dark orange (#a56d4f), dull, glabrous, with alternating concentric zones that are black (#000000) to dark orange (#ac7758); dense and radial fine wrinkles; ***margin*** dark red (#984b3e), acute, entire, wavy, and slightly incurved when dry; ***Context*** up to 1 mm, homogeneous, light grayish (#d4d9ce), and corky without black melanoid lines. ***Tubes*** up to 4 mm long, grayish orange (#d4c9b7), corky, and unstratified. ***Pores*** are 4–5 per mm, long oval to long polygon, dark grayish (#ac9d90) when dry, changing to red when bruised, then quickly darkening when fresh, without discoloration and entire dissepiments when dry. ***Stipe*** is up to 8.9 cm long and 4 mm in diameter, cylindrical, hollow, slightly curved, dark grayish (#958377) to black (#000000), and fibrous to woody.

***Hyphal system trimitic***, with generative hyphae are 4–8 μm in diameter, hyaline, thin-walled, and with clamp connections; skeletal hyphae are 4–7 μm in diameter, pale yellow, thin- to thick-walled with a wide or narrow lumen to subsolid, arboriform, branched, and flexuous; binding hyphae are 1–3 μm in diameter, pale yellow, branched, and flexuous. All hyphae IKI–, CB–. Context darkened in KOH. ***Pileipellis*** hymeniderm, close arrangement appears as regular palisade, apical cells are 40–80 × 5–8 μm, long stick, pale yellow. ***Basidiospores*** are ellipsoid to subglobose, faint yellow, IKI– and CB+, with double and thin walls, exospore wall smooth, endospore wall features conspicuous pillars, (10.0–) 10.3–13.4 (–13.5) × (8.9–) 9.1–10.7 (–10.9) μm, L = 11.4 μm, W = 9.8 μm, Q = 1.16 (40/2). Under SEM, exospore wall has regular and continuous wart-like protrusions. Cystidia absent. ***Basidia*** broadly clavate to subglobose, thin-walled, 19–29 × 16–18 μm; ***Basidioles*** broadly clavate, hyaline, thin-walled, 20–24 × 16–20 μm.

##### Additional specimens examined.

China • Yunnan Province, Dehong Prefecture, on the ground with humus, alt. 1,492 m, 24.357600°N, 98.049762°E, Kai-Yang Niu, 7 August 2023, HKAS 144479.

##### Notes.

In multi-locus phylogenetic analysis, *S.aurantiacus* formed a sister clade with *S.bambusae*. However, *S.aurantiacus* differs from *S.bambusae* by having a dark orange pileus surface with a dark red margin, thinner context (1 mm), and larger pores (4–5 per mm) (Table 2).

**Table 2.** Morphological comparison of *Sanguinodermabambusae* sp. nov., *S.niger* sp. nov., and *S.aurantiacus* sp. nov., with their closest relatives in the combined phylogeny. The new species are shown in bold.

Species	Pileus	Stipe	Pores	basidiospores	Reference
***Sanguinodermaaurantiacus***	**funnel-shape, dark orange, dull, glabrous**	**centrally to laterally, cylindrical, slightly curved, dark grayish to black**	**4-5 per mm**	**10.3–13.4 × 9.1–10.7 μm**	**this study**
***S.bambusae***	**umbelliform, dark gray to black, dull, glabrous**	**central, slightly curved, grayish orange to black**	**6–9 per mm**	**10.3–13.8 × 8.7–10.9 μm**	**this study**
*S.dehongense*	flabelliform to reniform, grayish yellow, dull, glabrous	laterally, cylindrical, swollen at base, dark-grayish orange	3–4 per mm	9.3–10.8 × 8.4–9.8 μm	Niu et al. 2024
*S.laceratum*	flabelliform or reniform, dark cinnamon, dull, tomentose	laterally, cylindrical and hollow, dark cinnamon	2–3 per mm	10.5–12.3 × 9–10.5 μm	Sun et al. 2020
*S.leucomarginatum*	near orbicular, fawn to vinaceous gray or near black, dull, glabrous	laterally, cylindrical, clay buff to fawn	5–6 per mm	8.8–10.1 × 7.8–9 µm	Sun et al. 2022b
***S.niger***	**circular, black to dark grayish orange, dull, tomentose**	**centrally, cylindrical, curved, swollen at base, grayish to grayish orange**	**6–7 per mm**	**9.2–11.8 × 8.2–9.9 µm**	**this study**
*S.ovisporum*	reniform, dark moderate orange, dull, tomentose	laterally, cylindrical, slightly swollen at base, very dark grayish orange	3–5 per mm	7.7–8.5 × 5.6–6.9 μm	Niu et al. 2024
*S.sinuosum*	suborbicular to flabelliform, dark brown to ferruginous, dull, glabrous	centrally to laterally, cylindrical and flexuous, dark brown to ferruginous	2–3 per mm	12.5–13.7 × 9.1–10.8 µm	Sun et al. 2022a

*Sanguinodermainfundibulare* B.K. Cui & Y.F. Sun and *S.aurantiacus* have similar dark orange pilei, concentric rings, and pores (4–6 per mm). However, *S.infundibulare* has a tomentose pileus surface, thicker context (4–5 mm), shorter pileipellis cells (23–30 × 6–11 μm), and smaller spores (10.2–12 × 9–10.2 μm) (Sun et al. 2022a).

#### ﻿ Sanguinoderma bambusae

Taxon classificationFungiPolyporalesGanodermataceae

K.Y. Niu, S.M. Tang & Z.L. Luo sp. nov.

01626FA5-550A-5B4A-A950-FF53767E4AD3

Fungal Names: FN 572237

Figs 3 , 5c, d

##### Diagnosis.

*Sanguinodermabambusae* differs from *S.laceratum* by having a dark gray and glabrous pileus, relatively small pores, dissepiments that remain intact when dry, centrally stipitate, slightly curved, grayish-orange to black stipe.

**Figure 3.**
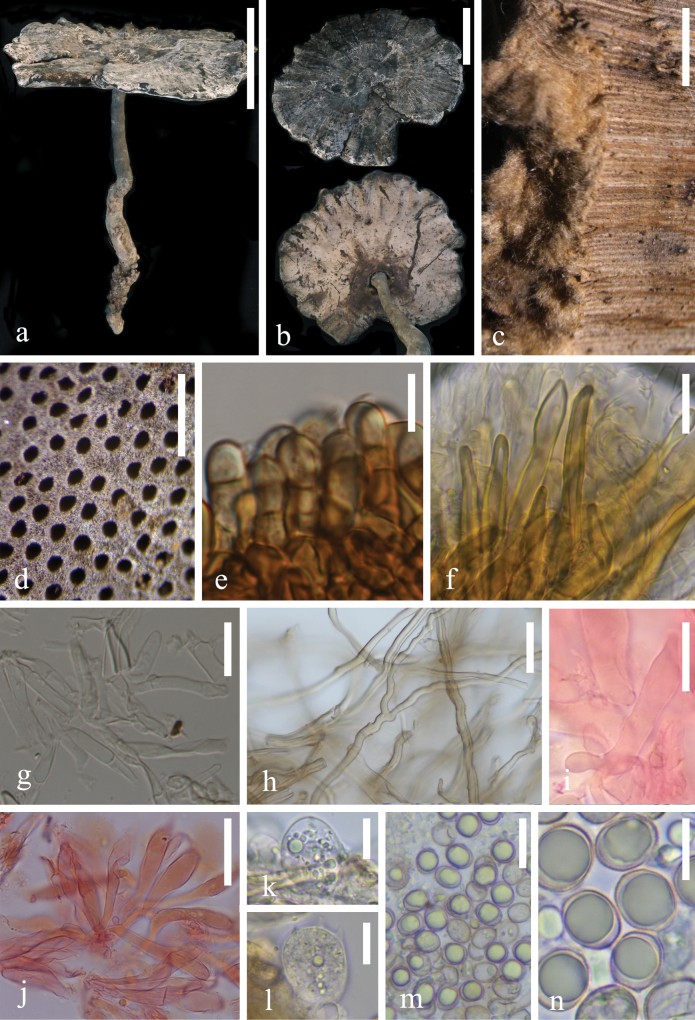
Morphological characteristics of *Sanguinodermabambusae* (HKAS 144474) **a, b** basidiomata **c** pileus cross-section **d** pores **e** pileipellis **f** skeletal hyphae from context **g** generative hyphae from tubes **h** binding hyphae from context **i–j** cystidia **k** basidioles **l** basidia **m, n** basidiospores. Scale bars: 10 cm (**a**); 5 cm (**b**); 2 mm (**c**); 0.5 mm (**d**); 10 μm (**e, k, n**); 20 μm (**f, i, m**); 15 μm (**g, l**); 30 μm (**h**); 35 μm (**j**).

##### Etymology.

The epithet “bambusae” refers to its growth on the ground in bamboo forests.

##### Holotype.

China • Yunnan Province, Dehong Prefecture, *Dendrocalamus* spp. forest humus, alt. 1,593 m, 24.754404°N, 98.235173°E, Kai-Yang Niu, 6 July 2023, HKAS 144474.

##### Description.

***Basidiomata*** annual, centrally stipitate, coriaceous to corky. ***Pileus*** umbelliform, up to 13.5 cm in diameter and 6 mm thick; ***pileus surface*** dark gray (#575757) to black (#000000), dull, glabrous, slightly dense, and radial fine wrinkles; ***margin*** dark gray (#5d5d5d), obtuse, entire, with sparse lacerated-like petal structures, slightly wavy and incurved when dry; ***Context*** up to 2 mm thick, homogeneous, slightly orange (#c2a37f), soft, and corky without black melanoid lines. ***Tubes*** up to 4 mm long, with the same color as the context, hard, and unstratified. ***Pores*** 6–9 per mm, oval to circular, grayish orange (#756961) when fresh, becoming red when bruised and then quickly darkening; without discoloration, dissepiments remain intact when dry. ***Stipe*** up to 27 cm long, 8 mm in diameter, central, cylindrical, hollow, slightly curved, grayish orange (#6b6252) to black (#000000), and fibrous to woody.

***Hyphal system trimitic*** with generative hyphae 3–6 μm in diameter, hyaline, thin-walled, and with clamp connections; skeletal hyphae 4–8 μm in diameter, pale yellow, thick-walled with a wide or narrow lumen to subsolid; binding hyphae 1–4 μm in diameter, pale yellow, flexuous, branched. All hyphae IKI– and CB+. Context darkened in KOH. ***Pileipellis*** trichoderm is a regular palisade, apical cells 5–7 × 20–32 μm, short clavate, and yellowish brown. ***Basidiospores*** broadly ellipsoid, light grey, IKI– and CB+ with double and thin walls; exospore wall is smooth; endospore wall features conspicuous pillars, (10.1–) 10.3–13.8 (–14.4) × (8.6–) 8.7–10.9 (–11.6), L = 11.6 μm, W = 9.6 μm, Q = 1.21 (n = 40/2). Under SEM, exospore wall has regular and continuous reticulate protrusions. ***Cystidia*** narrowly cylindrical to narrowly clavate, hyaline, thin-walled, and 44–50 × 5–9 μm. ***Basidia*** barrel-shaped to widely clavate, hyaline, thin-walled, and 25–26 × 16–18 μm. ***Basidioles*** elongated ellipse to ellipse, hyaline, thin-walled, and 20–22 × 14–18 μm.

##### Additional specimens examined.

China • Yunnan Province, Dehong Prefecture, on *Dendrocalamus* spp. forest humus, alt. 1,702 m, 24.731679°N, 98.255132°E, Kai-Yang Niu, 6 July 2023, HKAS 144473.

##### Notes.

In multi-locus phylogenetic analysis, *Sanguinodermabambusae* formed a sister clade with *S.ovisporum* K.Y. Niu, J. He & Z.L. Luo, and *S.laceratum* Y.F. Sun & B.K. Cui—all three species have been reported in Yunnan, China. Morphologically, *Sanguinodermabambusae* differs from *S.ovisporum* (Niu et al. 2024) and *S.laceratum* (Sun et al. 2020) by its centrally stipitate, dark gray pileus and smaller pores (6–9 per mm) (Table 2).

*Sanguinodermadehongense* K.Y. Niu, J. He & Z.L. Luo is morphologically similar to *S.bambusae* with an orange context and tubes, soft and corky without black melanoid lines, stipe length (21 cm), and slightly curved. However, *S.dehongense* differs by its larger pores (3–4 per mm), smaller basidia (8–11 × 9–13 μm), and basidiospores (9.3–10.8 × 8.4–9.8 μm) (Niu et al. 2024) (Table 2).

#### ﻿ Sanguinoderma niger

Taxon classificationFungiPolyporalesGanodermataceae

K.Y. Niu, S.M. Tang & Z.L. Luo sp. nov.

FACB5E65-607F-58BD-BB68-4D7F19AF2868

Fungal Names: FN 572238

Figs 4 , 5e, f

##### Diagnosis.

*Sanguinodermaniger* differs from *S.leucomarginatum* by having a tomentose, centrally stipitate pileus with dense and radial fine wrinkles, relatively large basidiospores, basidia, and basidioles.

**Figure 4.**
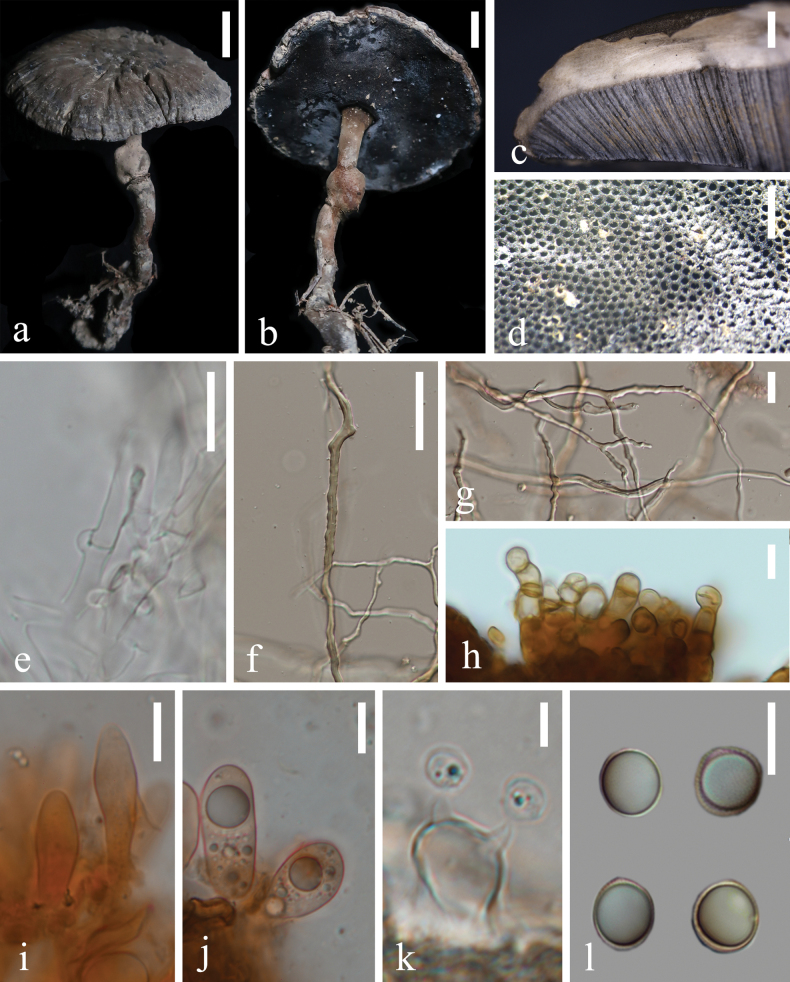
Morphological characteristics of *Sanguinodermaniger* (HKAS 144477) **a, b** basidiomata **c** pileus cross-section **d** pores **e** generative hyphae **f** skeletal hyphae **g** skeletal hyphae and binding hyphae **h** pileipellis **i** cystidia **j** basidioles **k** basidia **l** basidiospores. Scale bars: 1 cm (**a–c**); 1 mm (**d**); 10 µm (**e, h, i, j, l**); 40 µm (**f**); 15 µm (**g**); 5 µm (**k**).

##### Etymology.

The epithet “*niger*” refers to the black pileus.

**Figure 5.**
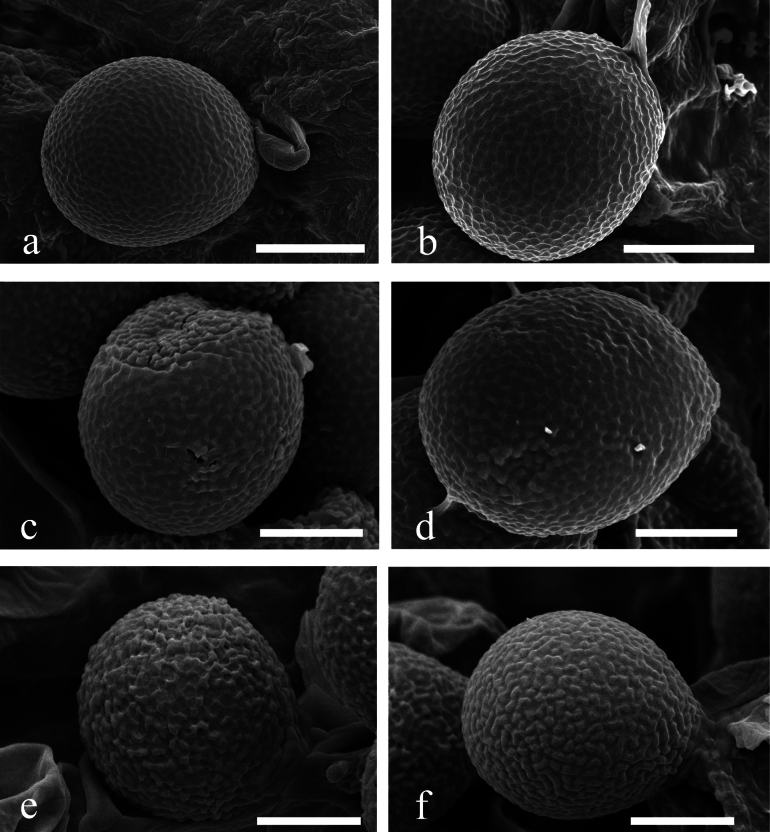
Scanning Electron Micrograph (SEM) of basidiospores of *Sanguinoderma***a, b***S.aurantiacus* (HKAS 144478) **c, d***S.bambusae* (HKAS 144474) **e, f***S.niger* (HKAS 144477). Scale bars: 5 μm (**a, b**); 3 μm (**c–f**).

##### Holotype.

China • Guizhou Province, Anshun City, on the ground covered with humus, alt. 1,087 m, 26.195484°N, 105.793283°E, Xing-Juan Xiao, 19 June 2024, HKAS 144477.

##### Description.

***Basidiomata*** annual, centrally stipitate, corky to woody, and hard. ***Pileus*** circular, up to 5 cm in diameter and 5 mm thick; ***Pileus surface*** black (#211f1f) to dark grayish orange (#5f5754), dull, tomentose, dense, and radial fine wrinkles; ***margin*** grayish (#a5a4aa), obtuse, entire, and does not curl when dried; ***Context*** up to 1 mm, homogeneous, grayish orange (#938d83) when dry, soft and corky without black melanoid lines. ***Tubes*** up to 4 mm long, dark grayish (#8b858f) when dry, hard, woody, and unstratified. ***Pores*** 6–7 per mm, spherical to elliptical, grayish yellow (#d9d2ba) when fresh, becoming red when bruised and then quickly darkening; without discoloration, dissepiments remain intact when dry. ***Stipe*** is up to 6 cm long and 4 mm in diameter, cylindrical, hollow, curved, swollen at base, grayish (#bbbbb9) to grayish orange (#8f796e), and fibrous to woody.

***Hyphal system trimitic***, with generative hyphae 3–4 µm in diameter, hyaline and thin-walled with clamp connections; skeletal hyphae 4–6 µm in diameter, pale grey to pale yellow and thick-walled with a wide to narrow lumen or subsolid, straight and little branched; binding hyphae are 2–4 µm in diameter, pale grey, thick-walled, branched, and flexuous; all hyphae IKI–, CB+. Context darkened in KOH. ***Pileipellis*** an irregular palisade; apical cells are 25–40 µm × 5–6 µm, tightly packed, narrow lumen, tightly packed together, thick-walled, and pale yellowish-brown, forming irregular palisade. ***Basidiospores*** subglobose to globose, pale gray, IKI–, and CB, with double and thick walls; the exospore wall is smooth; endospore wall features faint pillars, (8.9–) 9.2–11.8 (–12.6) × (8.0–) 8.2–9.9 (–10.0) µm, L = 10.8, W = 9.0, and Q = 1.19 (40/2). Under SEM, exospore wall has irregular and discontinuous wart-like protrusions. ***Cystidia*** narrowly utriform, hyaline, thin-walled, and 21–27 × 4–8 µm. ***Basidia*** broadly clavate, hyaline, thin-walled, and 20–23 × 10–13 µm. ***Basidioles*** obovoid to broadly clavate, with many small to large water droplets present, hyaline, thin-walled, and 17–21 × 9–11 µm.

##### Additional specimens examined.

China • Guizhou Province, Anshun City, on the ground covered with humus, 1,167 m, Xing-Juan Xiao, 19 June 2024, 26.191228°N, 105.826624°E, HKAS 144475; • *ibid*., 1,155 m, Xing-Juan Xiao, 19 June 2024, HKAS 144476.

##### Notes.

In the multi-locus phylogenetic analysis, *S.niger* formed a sister clade with *S.leucomarginatum*. However, *Sanguinodermaleucomarginatum* differs from *S.niger* (Sun et al. 2022b) by the lacks tomentose, white pileus surface, grayish-orange context with black melanoid lines, and longer cystidia (14–20 µm) (Table 2).

*Sanguinodermasinuosum* Y.F. Sun & B.K. Cui was originally described from Australia. It is similar to *S.niger* with circular pileus and centrally stipitate and has radial wrinkles on the pileus without concentric rings. However, *S.sinuosum* has a glabrous pileus, a pileus that is thicker (16 mm), with larger pores (2–3 per mm), absent cystidia and cystidioles, longer pileipellis (50–70 μm), and larger basidiospores (12.5–13.7 × 9.1–10.8 μm). Furthermore, *S.niger* clusters together with *Sanguinoderma* sp. 1 reported by Sun et al. (2022b), receiving good support (84/0.99). *Sanguinoderma* sp. 1 samples (Cui 11017 and HMAS 59720) were collected from Guizhou and Yunnan provinces in China, pileus concentric zones, and radial wrinkles. However, the mature basidiomata of *Sanguinoderma* sp. 1 (Cui 11017) were not obtained (Sun et al. 2022b); thus, these specimens have not been described.

## ﻿Discussion

In this study, based on phylogenetic analyses and morphological characteristics, three new species, *viz.*, *Sanguinodermaaurantiacus. S.bambusae* and *S.niger* are proposed.

*Sanguinodermaaurantiacus*, discovered in Dehong Prefecture, Yunnan, China, looks similar to the sympatric *S.concentricum* and *S.ovisporum*, all featuring an orange or near-orange pileus. However, stipes of *S.concentricum* and *S.ovisporum* are laterally attached, tubes of *S.ovisporum* are mostly desaturated dark orange and light gray near the pores, and the pileus margin of *S.concentricum* is lacerated-like petals (Table 2).

Sun et al. (2022b) regarded five clades as part of the *S.rugosum* complex, namely *S.rugosum*, *S.leucomarginatum*, and three undescribed species, because their basidiomata were immature. Our phylogeny analysis showed that *S.niger* clustered with *Sanguinoderma* sp. 1 and received good support (84/0.99).

**Figure 6.**
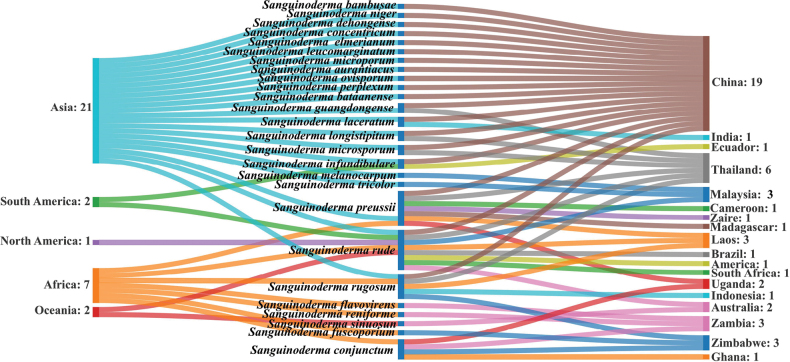
Global distribution of *Sanguinoderma* species.

*Sanguinoderma* is distributed across five continents and 18 countries (Fig. 6) (Hyde amd Wen 2018; Hapuarachchi et al. 2019; Sun et al. 2020, 2022a, 2022b; Peres et al. 2023). China has the highest diversity of *Sanguinoderma* species, followed by Thailand with six species (Fig. 6). A key to *Sanguinoderma* species in China is provided below. *Sanguinodermarude* is the most widely distributed species and has been reported in eight countries: America, Australia, Brazil, China, Laos, Malaysia, South Africa, and Thailand. These distribution patterns demonstrate the widespread presence of *Sanguinoderma* species.

### ﻿Key to accepted species of *Sanguinoderma* in China

**Table:** 

1	Pore dissepiments extremely thick	***S.microporum***
–	Pore dissepiments thin to distinctly thick	**2**
2	Pore dissepiments lacerate, tubes fascicular when dry	***S.laceratum***
–	Pore dissepiments entire, tubes unchanged when dry	**3**
3	Basidiospores are smaller (4.7–5.6 × 4.3–5.2 μm)	***S.microsporum***
–	Basidiospores are larger	**4**
4	Pores are smaller (6–9 per mm)	***S.bambusae***
–	Pores are larger	**5**
5	Tubes have two colors	***S.ovisporum***
–	Tubes have a single color	**6**
6	Pileus surface margin white to buff	***S.leucomarginatum***
–	Pileus surface margin not white to buff	**7**
7	Context is 1 mm thick	**8**
–	Context is more than 1 mm thick	**9**
8	Tubes more than 3 mm long	***S.aurantiacus***
–	Tubes less than 3 mm long	***S.preussii***
9	Pileus funnel-shaped	***S.infundibulare***
–	Pileus flat or umbelliform	**10**
10	Stipes is relatively thick (3 cm diameter)	**11**
–	Stipes is relatively thin (less than 3 cm diameter)	**12**
11	Basidiospores globose to subglobose (9.2–11.1 × 9–10.1 μm)	***S.elmerianum***
–	Basidiospores subglobose to broadly ellipsoid (11.5–14 × 10–12 μm)	***S.perplexum***
12	Pileus surface without concentric zones	***S.niger***
–	Pileus surface with concentric zones	**13**
13	Basidia are longer (30–40 μm)	***S.bataanense***
–	Basidia are shorter	**14**
14	Pileus tomentose	**15**
–	Pileus glabrous	**16**
15	Pileus suborbicular to umbelliform, dark yellowish brown to near black	***S.guangdongense***
–	Pileus orbicular to suborbicular, dark orange	***S.concentricum***
16	Context without resinous lines	**17**
–	Context with resinous lines	**18**
17	Pileus surface dark brown to ferruginous	***S.rude***
–	Pileus surface grayish yellow	***S.dehongense***
18	Context with two resinous lines	***S.rugosum***
–	Context with one resinous lines	***S.longistipitum***

Macrofungi, with their profound impact on forest ecosystem processes, stand out as pivotal contributors to the intricate balance and energy flow within forest ecosystems, in which they exhibit diverse morphological features in their basidiomata, and this group includes fungi known as polypores, corticioids, and hydnoids within aphyllophoroid fungi (Song et al. 2016; Sun et al. 2020; Wu et al. 2020; Zhao et al. 2023; Dong et al. 2024; Niu et al. 2024; Wang et al. 2024). The genus *Sanguinoderma* is a core group within macrofungi (Sun et al. 2022b). Similar to the difficulties encountered by Sun et al. (2022b) with the *S.rugosum* complex, we also observed a high proportion of immature basidiomata in our collections, which exhibited differences in macroscopic morphology. The immaturity of these specimens may be related to the species’ specific growth cycle or environmental requirements. In the future, it will be necessary to collect more specimens to gain a more comprehensive understanding of the *S.rugosum* complex.

*Sanguinoderma* grows mainly on forest floors, deciduous tree trunks, and various decayed stumps (such as *Litchi*, *Eucalyptus*, and *Acacia*) (Sun et al. 2020, 2022a, 2022b). Rarely, some species inhabit unique environments, such as *S.sinuosum*, which grows on sandy grounds in Australia (Sun et al. 2020). In this study, *S.bambusae* grew in *Dendrocalamus* sp. forest humus, representing a new habitat record for the *Sanguinoderma*. Bamboo forests have higher carbon storage than non-bamboo forests (Pambayun et al. 2023); macrofungi that acquire higher levels of carbon may develop larger fruiting bodies (Bässler et al. 2015). The significantly larger basidiomata of *S.bambusae* compared to other *Sanguinoderma* species in our samples suggest a possible link between carbon availability and fruiting body size, but further research is still required to confirm this.

## Supplementary Material

XML Treatment for Sanguinoderma aurantiacus

XML Treatment for Sanguinoderma bambusae

XML Treatment for Sanguinoderma niger

## ﻿Additional information

### Author ORCIDs

Kai-Yang Niu https://orcid.org/0009-0000-8479-890X

Feng-Ming Yu https://orcid.org/0000-0001-9133-8645

Lin Li https://orcid.org/0009-0000-8167-2965

Zong-Long Luo https://orcid.org/0000-0001-7307-4885

Song-Ming Tang https://orcid.org/0000-0002-6174-7314

### Data availability

All of the data that support the findings of this study are available in the main text.
